# Prenatal Exposure to Airborne Polycyclic Aromatic Hydrocarbons and Children’s Intelligence at 5 Years of Age in a Prospective Cohort Study in Poland

**DOI:** 10.1289/ehp.0901070

**Published:** 2010-04-20

**Authors:** Susan Claire Edwards, Wieslaw Jedrychowski, Maria Butscher, David Camann, Agnieszka Kieltyka, Elzbieta Mroz, Elzbieta Flak, Zhigang Li, Shuang Wang, Virginia Rauh, Frederica Perera

**Affiliations:** 1 Columbia Center for Children’s Environmental Health, Mailman School of Public Health, Columbia University, New York, New York, USA; 2 Department of Epidemiology and Preventive Medicine, Jagiellonian University, Krakow, Poland; 3 Polish-American Institute of Pediatrics, Jagiellonian University, Krakow, Poland; 4 Department of Analytical and Environmental Chemistry, Southwest Research Institute, San Antonio, Texas, USA; 5 Department of Biostatistics and; 6 Department of Population and Family Health, Mailman School of Public Health, Columbia University, New York, New York, USA

**Keywords:** air pollution, child, development, environmental, ETS, in utero, intelligence, prenatal, Poland, Raven

## Abstract

**Background:**

In this prospective cohort study of Caucasian mothers and children in Krakow, Poland, we evaluated the role of prenatal exposure to urban air pollutants in the pathogenesis of neurobehavioral disorders.

**Objectives:**

The objective of this study was to investigate the relationship between prenatal polycyclic aromatic hydrocarbon (PAH) exposure and child intelligence at 5 years of age, controlling for potential confounders suspected to play a role in neurodevelopment.

**Methods:**

A cohort of pregnant, healthy, nonsmoking women was enrolled in Krakow, Poland, between 2001 and 2006. During pregnancy, participants were invited to complete a questionnaire and undergo 48-hr personal air monitoring to estimate their babies’ exposure, and to provide a blood sample and/or a cord blood sample at the time of delivery. Two hundred fourteen children were followed through 5 years of age, when their nonverbal reasoning ability was assessed using the Raven Coloured Progressive Matrices (RCPM).

**Results:**

We found that higher (above the median of 17.96 ng/m^3^) prenatal exposure to airborne PAHs (range, 1.8–272.2 ng/m^3^) was associated with decreased RCPM scores at 5 years of age, after adjusting for potential confounding variables (*n* = 214). Further adjusting for maternal intelligence, lead, or dietary PAHs did not alter this association. The reduction in RCPM score associated with high airborne PAH exposure corresponded to an estimated average decrease of 3.8 IQ points.

**Conclusions:**

These results suggest that prenatal exposure to airborne PAHs adversely affects children’s cognitive development by 5 years of age, with potential implications for school performance. They are consistent with a recent finding in a parallel cohort in New York City.

Polycyclic aromatic hydrocarbons (PAHs), such as benzo[*a*]pyrene, are ubiquitous air pollutants released to ambient and indoor air from combustion sources such as coal-burning power plants, diesel- and gasoline-powered vehicles, home heating, and cooking and that are present in tobacco smoke and charred foods [Agency for Toxic Substances and Disease Registry (ATSDR) 1995]. Coal-burning power plants, home heating, traffic emissions, and secondhand smoke are the main contributors to airborne PAH levels in Poland (Choi et al. 2006).

Many studies indicate that the fetus and infant are more sensitive than adults to environmental toxicants including PAHs, lead, pesticides, and environmental tobacco smoke (ETS), because detoxification and DNA repair systems are immature and rates of cell proliferation are increased [National Research Council (NRC) 1993b; Perera et al. 2005; Whyatt and Perera 1995; World Health Organization 1986]. The central nervous system is particularly vulnerable during prenatal development (Rodier 2004). PAHs readily cross the placenta (Neubert and Tapken 1988; Perera et al. 2003).

PAHs have been shown to be neurodevelopmental toxicants in experimental studies (Saunders et al. 2006; Šrám and Binkova 2000; Wormley et al. 2004). Although the precise mechanisms by which they might affect the developing brain are not known, suggested mechanisms include endocrine disruption (Archibong et al. 2002; Bui et al. 1986; Takeda et al. 2004), binding to placental growth factor receptors (Dejmek et al. 2000), binding to the human Ah receptor to induce P450 enzymes (Manchester et al. 1987), DNA damage resulting in activation of apoptotic pathways (Metzer et al. 1995; Nicol et al. 1995; Wood and Youle 1995), and oxidative stress (Saunders et al. 2006). In addition, prenatal PAH exposures may affect epigenetic programming with neurologic consequences (Barker 2004; Perera et al. 2009a; Schwartz 2004; Wilson and Jones 1983).

A prospective cohort study of African-American and Latina mothers and children in New York City (NYC) that parallels the present study has reported that prenatal exposure to airborne PAHs is significantly associated with developmental delay at 3 years of age, as measured by the Bayley Scales of Infant Development (NRC 1993a; Perera et al. 2006), and with reduced IQ at 5 years of age, measured by the Wechsler Preschool and Primary Scale of Intelligence–Revised (WPPSI-R) (Perera et al. 2009b). In light of this evidence of neurodevelopmental effects in a multiethnic NYC population, in this analysis we evaluated the effects of prenatal airborne PAH exposure on a measure of child intelligence in a Caucasian population.

## Materials and Methods

### Krakow study population

This study is part of an ongoing, longitudinal investigation of the health effects of prenatal exposure to outdoor and indoor air pollution on infants and children in Krakow, Poland. As described previously (Jedrychowski et al. 2004), eligibility criteria included: nonsmoking women, ≥18 years of age, with singleton pregnancies; no current occupational exposure to PAHs or any other known developmental toxicants; no history of illicit drug use, pregnancy-related diabetes, or hypertension; and registration at a prenatal healthcare clinic in Krakow, their residence for at least 1 year preceding screening. A total of 505 pregnant (8–13 weeks) women fulfilled these criteria.

Full enrollment required providing prenatal questionnaire data, complete prenatal air monitoring data, and a blood sample at delivery from the mother and/or her newborn child. A total of 358 women were fully enrolled by these criteria, of whom 344 had valid airborne PAH data (meeting quality control criteria). We excluded 10 mother–child pairs whose cord or maternal blood cotinine levels registered > 25 ng/mL, above which active smoking during pregnancy is suspected (Vartiainen et al. 2002). Of the remaining 334 children, 214 children reached the age of 5 years by August 2009 and had complete Raven Coloured Progressive Matrices (RCPM) test results. Written informed consent was obtained from all mothers on their own behalf and for their child. The study was approved by the ethics committee of Jagiellonian University and by the institutional review board of the New York Presbyterian Medical Center.

### Prenatal interview

A 45-min questionnaire was administered by a trained interviewer during the second or third trimester of pregnancy to obtain demographic, health, and environmental data from the mothers. The questionnaire elicited information on ETS exposure during pregnancy (presence/absence of smokers in the household during pregnancy), dietary PAHs (frequency of consumption of broiled, fried, grilled, or smoked meat during pregnancy), and socioeconomic information related to income and education. Postnatal follow-up interviews were then administered to mothers every 6 months after birth to determine any changes in residence, exposure to ETS, and other health or environmental conditions.

### Personal air monitoring

To assess exposure to airborne PAHs, the women were personally monitored over a 48-hr period during the second (*n* = 253) or third (*n* = 100) trimester of pregnancy. During the day, they carried small backpacks holding personal air monitors and kept the monitors by their beds at night (Jedrychowski et al. 2004). As previously described (Camann and Whyatt 2001; Tonne et al. 2004), the polyurethane foam cartridges were analyzed at Southwest Research Institute in San Antonio, Texas, for concentrations of eight carcinogenic PAHs: benz[*a*]anthracene; chrysene/iso-chrysene; benzo[*b*]fluoranthene; benzo[*k*]fluoranthene; benzo[*a*]pyrene; indeno[1,2,3-*cd*]pyrene; dibenz[*a,h*]anthracene; and benzo[*g,h,i*]perylene. For quality control, we assessed each monitoring result for accuracy in flow rate, time, and completeness of documentation. Only samples meeting quality control criteria were included in the analysis. Each PAH measured by personal air monitoring was detectable in 100% of personal air samples with a wide range of concentrations. Because the eight airborne PAHs were highly intercorrelated (0.95 < Spearman’s *r* < 0.99) and to be consistent with the NYC and other studies (Perera et al. 2003), these PAHs were summed to provide a measure of total airborne PAHs, hereafter PAHs.

The use of personal monitoring was validated in this cohort in a subset of women (*n* = 80) who were simultaneously monitored for personal, indoor, and outdoor airborne PAHs. All three measurements were highly correlated (pairwise Spearman’s coefficients ≥ 0.84, *p* < 0.01) (Choi et al. 2008), which supported the use of personal monitoring to integrate indoor and outdoor exposure.

### Biological sample collection and analyses

After delivery, a cord blood sample was collected from the umbilical cord vein, and a venous blood sample was obtained from the women. Plasma cotinine and lead levels in cord and maternal blood and levels of 24 urinary PAH metabolites in child urine collected at the third year follow-up were measured at the U.S. Centers for Disease Control and Prevention (Atlanta, GA, USA) using high-performance liquid chromatography atmospheric-pressure ionization tandem mass spectrometry (cotinine) (Bernert et al. 1997), inductively coupled plasma mass spectrometry (lead) (Date and Gray 1989), and a combination of enzymatic deconjugation, automated liquid-liquid extraction, and gas chromatography/isotope dilution high resolution mass spectrometry (PAH metabolites) (Li et al. 2006), respectively. The PAH metabolites were creatinine adjusted to control for dilution.

### Neurodevelopmental testing

At the 5-year follow-up point, a trained research worker administered to each child the RCPM, a widely used age-adjusted nonverbal test of reasoning ability and intelligence based on figural materials or patterns (Raven et al. 1998). This instrument, which is listed as a cognitive test by the ATSDR, provides information on functioning in a number of cognitive domains such as visual-perceptual, language, praxis (performance), reasoning, and concept formation. Because it is reported to have minimal cultural bias and to be capable of evaluating the intellectual status of children exposed to toxic chemicals (Sattler 1986; Sizemore and Amler 1996), the RCPM has been used in a number of studies of environmental exposures in children in Poland and elsewhere (Szustrowa and Jaworowska 1992). Both the RCPM and the WPPSI-R, which was administered to the NYC cohort, are correlated with the standard test of intelligence called the Wechsler Intelligence Scale for Children (Raven et al. 1998). RCPM standard scores can be converted to IQ scores for estimates of intelligence levels (Counter et al. 2005; Raven et al. 1998), although they should not be interpreted as a measure of global intelligence, but of nonverbal intelligence (Counter et al. 2009). Here, to compare the Krakow and the NYC study results, we converted measured RCPM scores to IQ scores according to the RCPM manual (Raven et al. 1998).

Maternal intelligence was assessed using the Test of Nonverbal Intelligence–Third Edition (TONI-3), a language-free measure of general intelligence considered to be relatively free of cultural bias (DeMauro 2001).

### Model and covariates

Two hundred fourteen participants were included in the primary analysis presented here. A subset of 171 of the participants had available data on maternal intelligence (TONI-3) that were included as a covariate in a separate model. Missing covariates or test information occurred as a result of loss to follow-up or lack of a biological specimen for biomarker analysis.

Multiple linear regression models were used to estimate the association between prenatal airborne PAH exposure and the RCPM score (modeled as a continuous variable) as a measure of child IQ. Total airborne PAH exposure was modeled both as a dichotomous measure (cut at the median = 17.96 ng/m^3^ and referred to as high/low in the tables) and as a continuous measure. The continuous PAH data were ln-transformed [Ln(PAH) in the tables], because the distribution of airborne PAH concentrations was markedly skewed.

A number of covariates were selected *a priori* for inclusion in the model, based on the literature and our own prior data. These included maternal report of ETS exposure in the household during pregnancy, sex of the child, and maternal education (completed/did not complete 12 years of schooling). The latter was used as a proxy for socioeconomic status (Jedrychowski et al. 2009b). Because maternal intelligence (continuous TONI-3) is a known correlate of child cognitive development (Kagan and Moss 1959; Noble and McCandliss 2005) but was available only in a subset of the children (*n* = 171), it was included with the other covariates in a secondary analysis.

Lead and dietary PAHs were not significant predictors of RCPM scores (*p* < 0.1 in univariate regression analysis) and thus were not included in our final model. Because prenatal PAH exposure was found previously to be associated with reduced birth weight and head circumference in this cohort (Choi et al. 2008), we evaluated their potential as intermediate variables by including them in separate models. We also adjusted for postnatal PAH exposure using individual creatinine-adjusted PAH metabolites in urine collected at 3 years of age and a measure of postnatal residence change as an indicator of possible change in exposure to airborne PAHs after birth. We further adjusted for maternal report of postnatal exposure to ETS in the home. We also checked for effect modification by trimester of PAH monitoring, PAH monitoring season, or season of birth by including terms for the interaction between PAH and trimester of PAH monitoring, between PAH and PAH monitoring season, and between PAH and season of birth in separate models.

All effect estimates and *p*-values (αset at 0.05) were generated using SAS (version 9.1.0.3; SAS Institute Inc., Cary, NC, USA).

## Results

The average concentration of total airborne PAHs (of adequate monitoring quality) in personal air samples was 39.5 ± 48.1 ng/m^3^, with a median of 17.96 ng/m^3^ (*n* = 344). Among all the children tested for RCPM at the 5-year follow-up (*n* = 329), the average RCPM score was 21.8 ± 4.1.

Table 1 compares the basic demographic characteristics of fully enrolled subjects having prenatal airborne PAH monitoring data and RCPM data at 5 years of age with fully enrolled subjects having PAH monitoring data but no RCPM. The characteristics of the two groups did not differ significantly except for newborn birth weight and maternal age. On average, the children with RCPM data were 133.2 g lighter at birth, and mothers were older by 1 year. The differences in the means are modest but statistically significant.

Correlations between airborne PAHs and ETS exposure, between airborne PAHs and dietary PAHs, and between airborne PAHs and maternal or cord cotinine were examined using Spearman rank-order correlation. None was found to be significant, either in the Krakow cohort as a whole or the subset studied here (*n* = 214) (Table 2). As expected, ETS exposure during pregnancy was significantly correlated with maternal and cord cotinine levels both in the entire cohort and in the present subset, supporting self-reported ETS as a reliable measure of ETS exposure. ETS exposure and dietary PAHs were correlated in the present subset (*n* = 214) but not in the larger cohort.

High prenatal airborne PAH exposure levels were associated with a significant, albeit modest, reduction in child intelligence in models with either dichotomous or Ln-transformed continuous variables for PAH exposure (Table 3). The estimated effect of prenatal airborne PAH was significant after adjusting for prenatal ETS in the home and other potential confounders. The inverse relationship between airborne PAH level and RCPM score remained after adjusting for trimester of monitoring (second or third).

Prenatal ETS in the home was a significant predictor of RCPM score, as shown in Table 3. Excluding prenatal ETS from the model did not materially alter the estimated effect size or *p*-value of PAH on child RCPM score [β= −1.3, *p* = 0.03 in the PAH high/low model; β = −0.5, *p* = 0.03 in the Ln(PAH) model after excluding ETS (*n* = 214)], indicating that prenatal ETS exposure is not a potential confounder of this association.

In the smaller subset having data for maternal intelligence (*n* = 171), the estimated effect of PAH exposure remained consistent and significant [β = −1.4, *p* = 0.04 for PAH high/low; β = −0.6, *p* = 0.05 for continuous Ln(PAH)]. Prenatal exposure to ETS in the home and maternal intelligence were significant or borderline-significant covariates in this model (data not shown).

Neither lead nor dietary PAHs was a significant predictor of RCPM scores (< 0.1) when included individually in the model. Lead (computed as a Ln-transformed variable) was not a significant predictor in the model after controlling for prenatal ETS exposure in the home, sex of the child, and maternal education; the effect of prenatal airborne PAH exposure remained significant with lead included in the model. The dietary route of exposure to PAHs was not a significant contributor to the effect of airborne PAHs, which remained significant after including dietary PAH in the model. The magnitude of the association between PAHs and RCPM was unchanged when either lead or dietary PAH exposure was included in the model.

Including birth head circumference or birth weight separately in the model did not alter the estimated effect of airborne PAH [adjusted for birth head circumference: β = −1.4, *p* = 0.02 for PAH high/low; β = −0.6, *p* = 0.02 for continuous Ln(PAH), *n* = 214; adjusted for birth weight: β = −1.3, *p* = 0.03 for PAH high/low; β = −0.5, *p* = 0.02 for continuous Ln(PAH), *n* = 214].

With respect to postnatal exposure to PAHs, we found no effect of postnatal urinary PAH metabolites on the magnitude of the effect of PAHs on RCPM. Twenty-three percent of families changed neighborhood of residence in the child’s first 3 years of life, with a likely though unmeasured change in airborne PAH exposure. Adjusting for change in neighborhood of residence did not alter the strength of the inverse association found between prenatal airborne PAHs and RPCM score. Controlling for postnatal exposure to ETS in the home (22% of mothers reported exposure during at least one of the 10 follow-up interviews given between birth and the child’s follow-up at 5 years of age) did not alter the estimated effect of PAHs.

There was no evidence of an interaction between trimester of PAH monitoring, PAH monitoring season, or season of birth. The *p*-values of interaction terms were > 0.38 in the models with dichotomized PAH and > 0.25 in the models with ln-transformed PAH.

Finally, to better compare the Krakow cohort with the NYC cohort, we restricted analysis to the Krakow participants within the common PAH exposure range seen in Krakow and NYC (0.27–44.81 ng/m^3^) and to the subset of women for whom data on maternal intelligence were available (so that the models would be directly comparable). In this comparison, the estimated effect sizes in both the dichotomous and continuous models were similar to those observed in the entire Polish cohort (over the full exposure range: 1.8–272.2 ng/m^3^) (Table 4).

## Discussion

We have found that higher prenatal exposure to airborne PAHs is associated with a modest and statistically significant reduction in scores on a test of nonverbal child intelligence in a sample of 5-year-old children of nonsmoking mothers from Krakow, Poland, after controlling for confounding variables. Children in the high-exposure group (> 17.96 ng/m^3^) had RCPM scores that were, on average, 1.4 points lower (*p* = 0.02) compared with less-exposed children (≤17.96 ng/m^3^), corresponding to an estimated 3.8-point average decrease in IQ points. The relationship between prenatal airborne PAH and intelligence at 5 years of age remained significant after controlling for postnatal exposure to PAHs and ETS in the home.

Because the Home Observation for Measurement of the Environment (HOME) Inventory—a measure of the child’s proximal caretaking environment that can confound a study on neurodevelopment (Bradley et al. 1996)—is not widely used in central Europe, it was not administered in the Polish cohort as in the parallel NYC cohort study; this constitutes a limitation of this study. However, we included maternal education in our models to partially account for the important role of the mothers in stimulating the child (Jedrychowski et al. 2009a; Kagan and Moss 1959; McAskie and Clarke 1976; Noble and McCandliss 2005). Because of the concern that this dichotomized variable (completed vs. did not complete 12 years of schooling) may itself not be sensitive enough to control for differences in the home environment and account for potential confounding of PAH effects by maternal education, we also used total years of education completed by the mother as a continuous variable and found no differences in the results. We also found that personal airborne PAH levels were not correlated with total years of education completed by the mother (*r* = −0.038, *p* = 0.488, *n* = 333), mitigating concern that unmeasured differences in socioeconomic status may have confounded our findings on PAH exposure. Maternal intelligence was available for a subset of 171 women and was significantly associated with child IQ. After adjustment for maternal intelligence, the effect of prenatal PAH exposure was significant (*p* = 0.0036 for high/low PAH and *p* = 0.039 for continuous PAH).

Prenatal ETS in the home was significantly associated with child IQ but did not confound the association between airborne PAHs and child intelligence. The association between prenatal exposure to ETS and deficits in early cognitive functioning has been established previously (Eskenazi and Castorina 1999). Prior research in the same Krakow cohort showed that cotinine levels measured in plasma of newborns were significantly higher than in the blood of the mothers, indicating that the fetus may be less able to detoxify this substance (Jedrychowski et al. 2007). Prenatal ETS was not correlated with airborne PAHs (Table 2), consistent with the analysis of Choi and colleagues, which showed that all eight speciated PAHs monitored have outdoor sources identified with coal combustion, although some are also related to ETS (Choi et al. 2006).

Transplacental exposures to PAHs have been linked to decrements in head circumference, birth weight, and birth length (Dejmek et al. 2000; Perera et al. 2003; Whyatt et al. 1998). These decrements have potential longer-term implications for producing lower cognitive functioning and poorer school performance in childhood (Hack et al. 1991). However, in this analysis, neither the head circumference nor birth weight of the newborn predicted intelligence at 5 years of age.

Relying on a single measurement of prenatal air for our exposure matrix is limited. However, because measurements during the second and third trimesters were correlated (Choi et al. 2008), we considered the single monitoring time point to be a reasonable indicator of prenatal exposure via inhalation over the last two trimesters of pregnancy. Despite known seasonal variation in air pollution levels related to heating with coal in Krakow (Choi et al. 2008), the potential effect of season on our results was mitigated by the fact that monitoring was evenly distributed across seasons (*n* = 86, 81, 85, and 92 mothers monitored in spring, summer, fall, and winter, respectively). We also considered the limitation of using a 48-hr monitoring period to represent a longer continuous exposure period. However, we found no effect modification by trimester of PAH monitoring, PAH monitoring season, or season of birth on any of our findings. We also did not make the assumption that the second and/or third trimesters of pregnancy are the most vulnerable periods with respect to brain development. The first trimester may be equally or more important.

To our knowledge, there has only been one prior epidemiologic study reporting an inverse association between prenatal exposure to PAH and child intelligence at 5 years of age, and that study is from our center’s ongoing cohort study in NYC (Perera et al. 2009b). By design, all variables were measured similarly in NYC and Krakow except for the neurodevelopment assessment tool used (RCPM in Krakow vs. the WPPSI-R score in NYC). The NYC study included African Americans and Dominican Americans; the Krakow study participants were Caucasians. Data on the home caretaking environment were available only in NYC. A further limitation of the Krakow study is the fact that the data on maternal intelligence were available only for a subset of the participants.

Nonetheless, the results of the two cohort studies, one in African Americans and Dominicans and the other in Caucasians, are generally consistent, both across the full range of exposure and restricting to the common, lower range. To compare the two different studies, we standardized the exposure effect sizes (β/SE) and found that they were similar in the two cohorts at age 5 years: −2.06 [95% confidence interval (CI), −4.02 to −0.1] on RCPM in Krakow (entire range) compared with −2.63 (95% CI, −4.59 to −0.67) for Full Scale IQ in the NYC cohort (Perera et al. 2009b). Restricting to the common, lower-exposure range, the standardized exposure effect size (β/SE) was −2.56 (95% CI, −4.52 to −0.6) on RCPM in Krakow, indicating general consistency of results across these two different cohorts at 5 years of age. Because the NYC and Krakow cohorts represent a wide range of exposures experienced in many other urban areas, study results in Krakow and NYC are relevant to other populations worldwide.

As noted, there are a number of limitations in this study. They include the single 48-hr prenatal monitoring, the lack of HOME Inventory data, and the fact that although the prenatal ETS questionnaire data were validated by cotinine, the estimate of postnatal ETS was based on interviews alone.

In summary, these results indicate that airborne PAHs may adversely affect children’s cognitive development at 5 years of age, with potential implications for school performance. Our findings are of concern because RCPM scores measured during the preschool period have been shown to correlate with academic achievement later in life (Balboni et al. 2010; Raven 2000). We are continuing to follow this cohort to determine longer-term effects of prenatal exposure on behavioral outcomes and academic readiness.

## Figures and Tables

**Table 1:** Subset with airborne PAH monitoring data and RCPM data (n = 219)^a^ versus subset with airborne PAH monitoring data but missing RCPM data in the Krakow cohort (n = 139).

		Subset with airborne PAH monitoring and RCPM		Subset with airborne PAH monitoring but missing RCPM	
Variable	*p*-Value^b^	Mean ± SD	*n*^c^	Mean ± SD	*n*^d^
Continuous					
Total valid airborne PAH exposure (ng/m^3^)	0.65	39.62 ± 48.49	219	39.39 ± 47.70	125
Dietary exposure to PAHs during pregnancy^e^	0.31	42.49 ± 5.65	219	43.14 ± 6.27	139
Cord blood cotinine level (ng/mL)	0.49	2.04 ± 11.52	219	3.29 ± 15.56	138
Cord blood lead level (μg/dL)	0.59	1.60 ± 1.19	210	1.51 ± 0.78	129
Cord blood mercury level (μg/dL)	0.43	1.07 ± 0.64	161	1.16 ± 0.79	106
Gestational age (days)	0.07	39.26 ± 1.73	219	39.51 ± 1.63	139
Birth weight (g)	0.01	3368.49 ± 508.38	219	3501.65 ± 474.22	139
Birth height (cm)	0.76	54.44 ± 3.06	219	54.54 ± 3.10	139
Head circumference at birth (cm)	0.21	33.81 ± 1.54	219	34.01 ± 1.47	139
Maternal age (years)	0.01	28.50 ± 3.59	219	27.49 ± 3.73	139
Maternal height (cm)	0.18	164.69 ± 5.71	219	165.52 ± 5.59	139
Maternal prepregnancy weight (kg)	0.95	58.44 ± 8.80	219	58.50 ± 9.32	139
Maternal TONI-3	0.23	33.48 ± 9.07	176	31.42 ± 9.71	31
Maternal blood cotinine level at birth (ng/mL)	0.97	1.68 ± 10.09	219	2.99 ± 14.34	138
Maternal blood lead level at birth (μg/dL)	0.79	1.92 ± 0.83	209	1.86 ± 0.66	136

Categorical	*p*-Value^f^	Proportion	*n*	Proportion	*n*

Caucasian race	1.00	1.00	219	1.00	139
Maternal education (proportion graduated high school, i.e., 12 years of schooling)	0.47	0.91	219	0.88	139
ETS in the home (yes = 1, no = 0)	0.52	0.22	219	0.25	139
Exposure to alcohol during pregnancy (yes = 1, no = 0)	0.18	0.60	219	0.67	139
Sex of the child (proportion female)	0.52	0.52	219	0.48	139

a Among these 219 mother–child pairs with valid PAH monitoring data and complete RCPM data, five pairs had blood cotinine levels > 25 ng/mL and were not included in the sample size of 214 presented in our final model (see Table 3). Mother–child pairs included in Table 1 were not restricted based on blood cotinine level because we thought this might bias our comparisons.

b *p*-Values were generated by two sample *t*-tests, except for cord blood cotinine level, maternal test of nonverbal intelligence, maternal blood cotinine level at birth, and gestational age, which were not normally distributed, and for which nonparametric Wilcoxon tests were therefore used.

c If the sample size is < 219, some subjects are missing data on the variable in question.

d If the sample size is < 139, some subjects are missing data on the variable in question. In the case of PAH exposure levels, only 125 of 139 had valid PAH data; i.e., meeting quality control standards.

e Estimated by the frequency of intake of PAH-containing foods during pregnancy, as reported in the prenatal questionnaire by the mother.

f *p*-Values were generated by Fisher’s exact test.

**Table 2:** Spearman correlations [correlation coefficient *r; p*-value in the Krakow cohort (*n*)].

	ETS	Maternal cotinine	Cord cotinine	Total airborne PAHs
Fully enrolled participants with adequate monitoring data				
Dietary PAH	0.09, 0.11 (344)	−0.02, 0.70 (343)	0.03, 0.60 (343)	0.06, 0.25 (344)
ETS	—	0.48, < 0.01 (343)	0.47, < 0.01 (343)	−0.05, 0.38 (344)
Maternal cotinine	—	—	0.85, < 0.01 (343)	0.08, 0.15 (343)
Cord cotinine	—	—	—	0.03, 0.53 (343)

Subset of children included in the present analysis (*n* = 214)^a^				
Dietary PAH	0.19, 0.01	−0.04, 0.57	−0.04, 0.54	0.05, 0.46
ETS	—	0.48, < 0.01	0.46, < 0.01	−0.05, 0.50
Maternal cotinine	—	—	0.99, < 0.01	0.02, 0.75
Cord cotinine	—	—	—	0.04, 0.60

a This subset excludes subjects with monitoring data not meeting quality control criteria, with maternal or cord cotinine levels > 25 ng/mL, and those for whom any of the following data are missing: child RCPM score, prenatal ETS in the home, sex of child, or maternal education.

**Table 3:** Association between prenatal exposure to airborne PAHs and RCPM score at 5 years of age in the Krakow cohort (*n* = 214).

	Dichotomous				Ln(PAH)		
*n* = 214	β	95% CI	*p*-Value	*n* = 214	β	95% CI	*p*-Value
PAH (high/low)	−1.36	−2.48 to −0.23	0.02	Ln(PAH)	−0.56	−1.00 to −0.11	0.02
ETS in the home^a^	−1.83	−3.3 to −0.36	0.02	ETS in the home	−1.86	−3.32 to −0.39	0.02
Sex of child^b^	0.57	−0.56 to 1.69	0.33	Sex of child	0.54	−0.58 to 1.67	0.35
Maternal education^c^	0.81	−1.32 to 2.93	0.46	Maternal education	0.75	−1.38 to 2.87	0.50

Two models are presented side by side, one with dichotomous PAH (high/low) and one with continuous Ln-transformed PAH [Ln(PAH)]. They both adjust for prenatal ETS in the home, sex of the child, and maternal education. After further including maternal intelligence (using continuous TONI-3 score), which was significantly associated with child intelligence in the model, the betas and *p*-values for PAH both high/low and Ln-transformed were similar and significant (*n* = 171). Lead, dietary PAHs, birth weight, birth head circumference, individual creatinine-adjusted PAH metabolites from urine, maternal report of postnatal exposure to ETS in the home, and whether the child changed residence postnatally were not significant predictors (*p* < 0.1) and were therefore not included in the final model presented here.

a Maternal report of smoke in the home during pregnancy: yes = 1, no = 0.

b Female = 1, male = 0.

c Graduated from high school, that is, completed 12 years of schooling: yes = 1, no = 0.

**Table 4:** Association between prenatal exposure to airborne PAHs and RCPM score at 5 years of age in the Krakow cohort including only subjects whose PAH exposure was within the common exposure range between the NYC and Krakow cohorts (0.27–44.81 ng/m^3^) (*n* = 150).

	Dichotomous				Ln(PAH)		
*n* = 150	β	95% CI	*p*-Value	*n* = 150	β	95% CI	*p*-Value
PAH (high/low)	−1.62	−2.98 to −0.26	0.02	Ln(PAH)	−1.24	−2.05 to −0.43	< 0.01
ETS in the home^a^	−2.07	−3.75 to −0.38	0.02	ETS	−1.91	−3.58 to −0.25	0.02
Sex of child^b^	0.63	−0.73 to 1.99	0.37	Sex of child	0.73	−0.61 to 2.07	0.28
Maternal education^c^	0.77	−2.02 to 3.57	0.59	Maternal education	1.00	−1.77 to 3.77	0.48

Two models are presented side by side, one with dichotomous PAH (high/low) and one with continuous Ln-transformed PAH [Ln(PAH)]. They both adjust for prenatal ETS in the home, sex of the child, and maternal education.

a Maternal report of smoke in the home during pregnancy: yes = 1, no = 0.

b Female = 1, male = 0.

c Graduated from high school, that is, completed 12 years of schooling: yes = 1, no = 0.
